# Modular signature of long non-coding RNA association networks as a prognostic biomarker in lung cancer

**DOI:** 10.1186/s12920-021-01137-0

**Published:** 2021-12-06

**Authors:** Albert Li, Wen-Hsuan Yu, Chia-Lang Hsu, Hsuan-Cheng Huang, Hsueh-Fen Juan

**Affiliations:** 1 Graduate Institute of Biomedical Electronics and Bioinformatics, National Taiwan University, Taipei, 10617 Taiwan; 2 Department of Medical Research, National Taiwan University Hospital, Taipei, 10002 Taiwan; 3 Institute of Biomedical Informatics, National Yang Ming Chiao Tung University, Taipei, 11221 Taiwan; 4 Department of Life Science, National Taiwan University, Taipei, 10617 Taiwan; 5 Center for Computational and Systems Biology, National Taiwan University, Taipei, 10617 Taiwan

**Keywords:** LncRNA association networks, Lung cancer, lncRNA modular prognostic biomarkers

## Abstract

**Background:**

Increasing amount of long non-coding RNAs (lncRNAs) have been found involving in many biological processes and played salient roles in cancers. However, up until recently, functions of most lncRNAs in lung cancer have not been fully discovered, particularly in the co-regulated lncRNAs. Thus, this study aims to investigate roles of lncRNA modules and uncover a module-based biomarker in lung adenocarcinoma (LUAD).

**Results:**

We used gene expression profiles from The Cancer Genome Atlas (TCGA) to construct the lncRNA association networks, from which the highly-associated lncRNAs are connected as modules. It was found that the expression of some modules is significantly associated with patient’s survival, including module N1 (HR = 0.62, 95% CI = 0.46–0.84, *p* = 0.00189); N2 (HR = 0.68, CI = 0.50–0.93, *p* = 0.00159); N4 (HR = 0.70, CI = 0.52–0.95, *p* = 0.0205) and P3 (HR = 0.68, CI = 0.50–0.92, *p* = 0.0123). The lncRNA signature consisting of these four prognosis-related modules, a 4-modular lncRNA signature, is associated with favourable prognosis in TCGA-LUAD (HR = 0.51, CI = 0.37–0.69, *p* value = 2.00e−05). Afterwards, to assess the performance of the generic modular signature as a prognostic biomarker, we computed the time-dependent area under the receiver operating characteristics (AUC) of this 4-modular lncRNA signature, which showed AUC equals 68.44% on 336th day. In terms of biological functions, these modules are correlated with several cancer hallmarks and pathways, including Myc targets, E2F targets, cell cycle, inflammation/immunity-related pathways, androgen/oestrogen response, KRAS signalling, DNA repair and epithelial-mesenchymal transition (EMT).

**Conclusion:**

Taken together, we identified four novel LUAD prognosis-related lncRNA modules, and assessed the performance of the 4-modular lncRNA signature being a prognostic biomarker. Functionally speaking, these modules involve in oncogenic hallmarks as well as pathways. The results unveiled the co-regulated lncRNAs in LUAD and may provide a framework for further lncRNA studies in lung cancer.

**Supplementary Information:**

The online version contains supplementary material available at 10.1186/s12920-021-01137-0.

## Background

Lung cancer is one of the leading causes of cancer mortality around the globe [1]. Although a few oncogenic alterations can be targeted by certain medications, which showed greater therapeutic efficacy than chemotherapy [2, 3], the prognostic biomarkers assessing lung cancer patients are rather limited currently [3]. Therefore, further exploration in biomarkers that assess the prognosis of lung cancer patients is warranted.

LncRNAs, transcripts with length greater than 200 nucleotides [4], regulate gene expression through various mechanisms, including cis- [5–7], trans-[8, 9], and post-transcriptional regulation. Aberrant lncRNA regulations were found in various cancers [10]. These perturbations in transcriptome can be revealed by lncRNA-mRNA co-expression networks, resulting in the discovery of several key lncRNAs and mRNAs that contributed to cancer progression [11, 12]. Subsequent in silico and clinical studies further suggested that multiple lncRNAs can be integrated into singular signatures to better predict cancer patients’ survival [13–16], tumour relapse [17, 18] and response to immunotherapy [15]. Given that the underlying biological explanation of combining multiple lncRNAs into one signature may still be unclear, it was speculated that functionally similar lncRNA may co-appear within cells. Therefore, identifying highly associated lncRNAs and their roles in LUAD is crucial.

A lncRNA-mRNA co-expression network is a bi-partite network. The association between each lncRNA can be measured in a lncRNA-mRNA bi-partite network via computing the association index, a quantitative metric measuring the similarity between two lncRNAs according to the overlapping correlated mRNAs. By constructing the lncRNA association networks, similar lncRNAs would be clustered as modules [19]. Functions of the lncRNA modules can then be deduced by the module-correlated mRNAs under the norm of ‘guilt by association’ [20].

However, our understanding regarding the associated lncRNAs in LUAD has been primitive. The roles of LUAD-specific modules are to be confirmed. Furthermore, whether lncRNA modules signature can be a decent prognostic biomarker needs further investigation. Hence, we postulated that lncRNA modules are composed of co-regulated lncRNAs, and these modules play crucial roles in LUAD. To validate the hypothesis, we gathered gene expression profile from TCGA-LUAD project, constructed the lncRNA association networks, deduced impact of modules on patients’ survival, and analysed biological roles of these lncRNA modules. In this study, we provided a module-based approach to uncover a novel biomarker in LUAD.

## Results

### Overview of the analytical pipeline

**Fig. 1:**
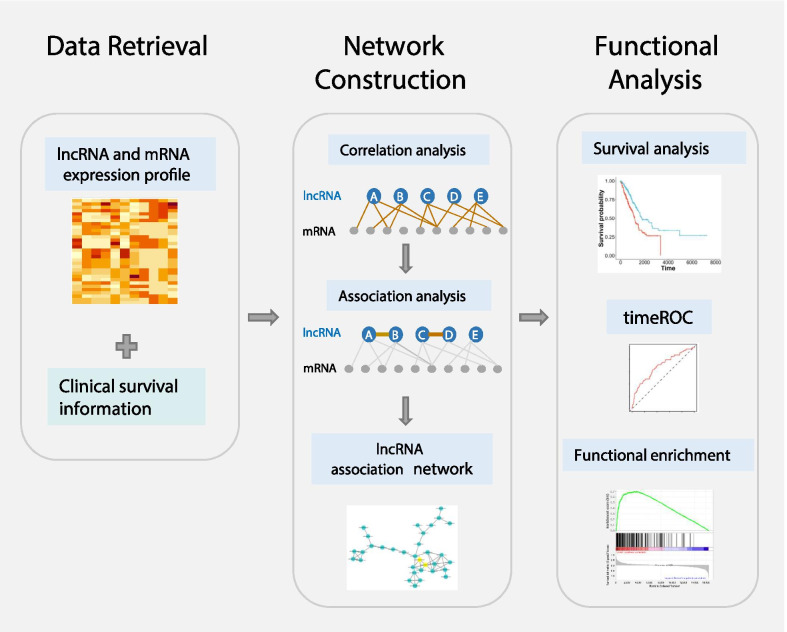
The flowchart of prognostic lncRNAs module discovery in LUAD. The expression profile of lncRNA and mRNA as well as clinical data were retrieved from TCGA. To determine the lncRNA-correlated mRNAs, the lncRNA-mRNA correlation was quantified by SCC and MR. The lncRNA association was computed by PCC. The lncRNA pairs with PCC > 0.7 were selected to construct the lncRNA association networks. LncRNAs that affect prognosis were selected and pooled to evaluate the impact lncRNA modules on survival. To evaluate the performance of generic modular lncRNA signature, we conducted time-AUC analysis. Finally, functional enrichment analysis was used to reveal potential mechanisms that lncRNA modules involved in. LUAD, lung adenocarcinoma; TCGA, the cancer genome atlas; SCC, Spearman’s rank correlation coefficient; MR, mutual rank; PCC, Pearson correlation coefficient

We proposed a computational pipeline (Fig. 1) to construct LUAD-specific lncRNA association networks and identify prognosis-related lncRNA modules. Firstly, the correlation between lncRNA and mRNA was quantified with Spearman correlation coefficient (SCC), which was then transformed to mutual rank (MR) index. The mRNAs within top- and bottom- MR index were selected as positive- and negative- correlated mRNAs respectively. Second, the association between each lncRNA was quantified by the association index. Next, we selected lncRNA pairs with top-scoring association index and constructed lncRNA association networks. Finally, we assessed roles of lncRNA modules in LUAD through survival analysis, time-dependent AUC, and functional enrichment analysis.

### Construction of the lncRNA association networks

**Fig. 2:**
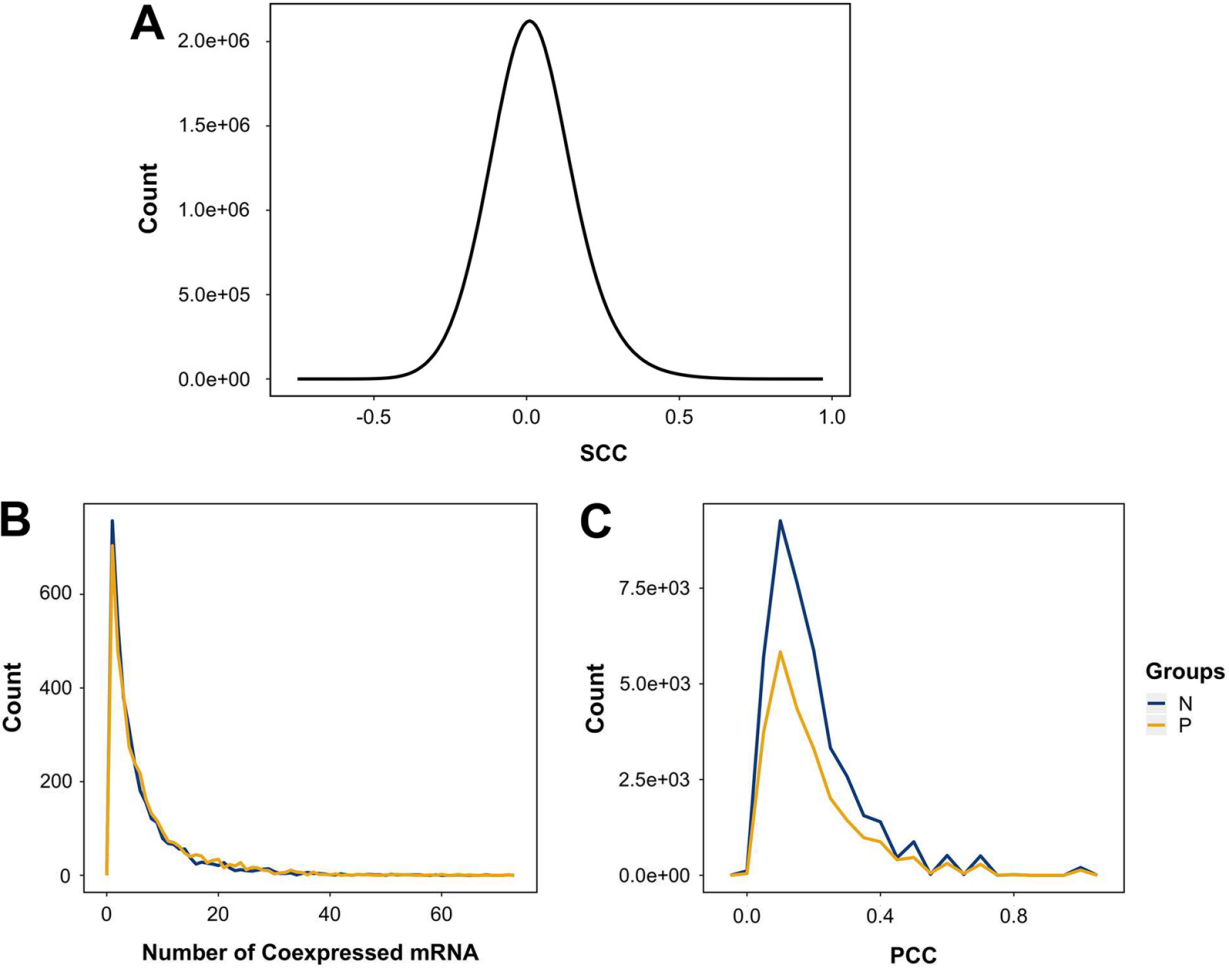
Identification of correlated lncRNA-mRNA pairs and associated lncRNAs. **A** The distribution of the SCCs of lncRNA-mRNA pairs. **B** The distribution of the number of correlated mRNAs per lncRNA. The lncRNA-positively correlated mRNAs are defined as top 0.1% of mutual rank index (yellow) of lncRNA-mRNA pairs; the lncRNA-negatively correlated mRNAs are defined as bottom 0.1% of mutual rank index (blue) of lncRNA-mRNA pairs. **C** The PCC between each lncRNA was calculated considering the number of their correlated mRNAs. The distribution of PCC according to the lncRNA-positively correlated mRNAs (blue) and lncRNA-negatively correlated mRNAs (yellow) were plotted. N, measurement according to lncRNA-negatively correlated mRNAs; P, measurement according to lncRNA-positively correlated mRNAs; SCC, spearman’s rank correlation coefficient

**Fig. 3:**
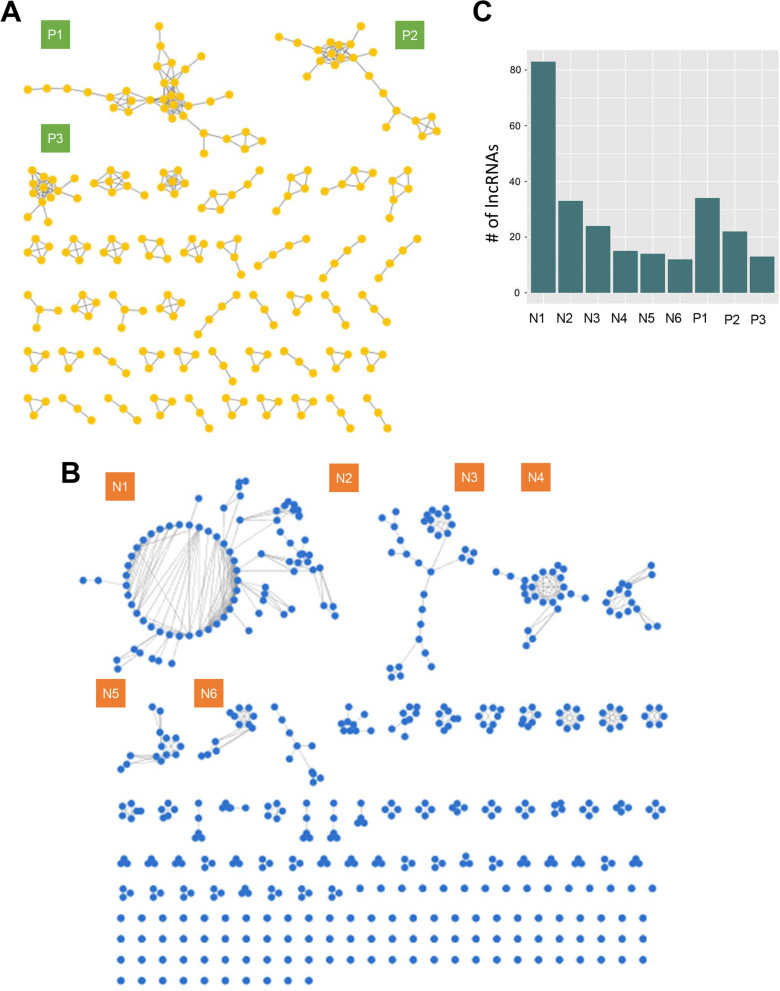
The lncRNA association networks. **A** The lncRNA-lncRNA pairs with PCC greater than 0.7 were selected to construct the positive lncRNA association network (PLAN). The calculation of PCC is based on the mRNAs that are positively correlated with lncRNAs. Modules P1-P3 (green square) are selected for following analyses. **B** As the above method, we measured the PCC based on the mRNAs that are negatively correlated with lncRNAs. The lncRNA pairs with PCC greater than 0.7 were collected to construct the negative lncRNA association network (NLAN). N1-N6 are modules selected for following analyses. **C** The number of nodes in each selected module

The gene expression profile of 553 LUAD samples were obtained from TCGA. After pre-processing, 4342 lncRNAs and 16,618 mRNAs remained. We quantified the correlation between lncRNAs and mRNAs with SCC (Fig. 2A). To identify the lncRNA-correlated mRNAs, SCCs were transformed to MR index [21]. The mRNAs with top and bottom 0.1% of MR index ($MR \le \sqrt {4342{ } \times { }16619} \times 0.001 \cong 8.49$) were selected as the lncRNA- positively or negatively correlated mRNAs. The number of co-expressed mRNA of each lncRNA was shown in Fig. 2B. The mRNAs that are highly correlated with lncRNAs were chosen to calculate the association index between lncRNAs (Fig. 2C). Several association indices are popular and commonly used, such as Jaccard index. We used Pearson Correlation Coefficient (PCC) in this study because PCC can group the associated genes (AUC = 0.903) and can best separate highly co-expressed gene pairs from the others (AUC = 0.750) [20]. We collected the lncRNA-lncRNA pairs having PCCs > 0.7 and constructed the lncRNA association networks (LAN). The LAN constructed from the positively or negatively correlated mRNAs were coined as positive lncRNA association network (PLAN) or negative lncRNA association network (NLAN), respectively (Fig. 3).

### Characteristics of the lncRNA association networks

**Fig. 4:**
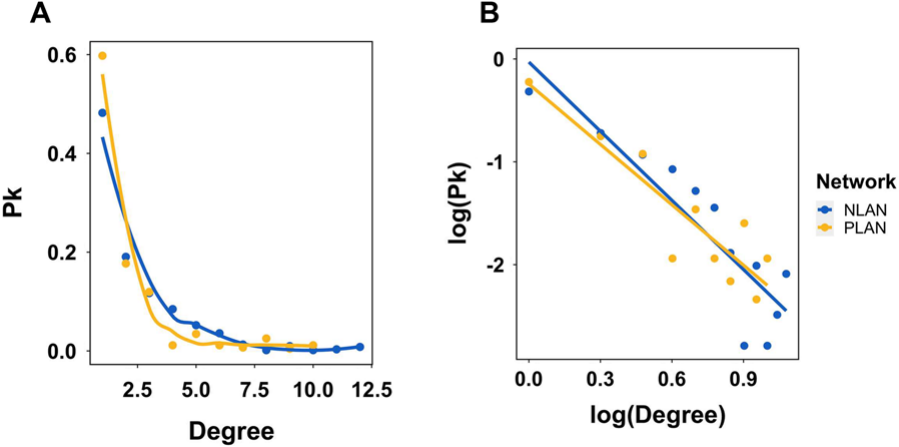
Identification of the scale-free property of the lncRNA association networks. **A** The degree of each node in the networks were plotted against the degree density (pk). The fitted curve showed power law distribution. **B** The value in both axes underwent logarithm transformation. The fitted regression lines were significant, with *p* = 6.51e−05 in NALN and *p* = 0.000292 in PLAN respectively

To verify the scale-free property of generic lncRNA association networks, we inspected the relation between the degree (*k*) of nodes and the degree density (*pk*) (Fig. 4A). The power law distribution was revealed when plotting *k* against *pk*. Further, when taking the logarithm on both axes, the regression line significantly fit the data points, with the slope coefficient = − 1.96, *p* value = 0.000292 on PLAN; the slope coefficient = − 2.24, *p* value = 6.51e-05 on NALN. (Fig. 4B). The above results suggested that the lncRNA association networks were non-random, and had the scale-free property. We selected modules with nodes no less than 12 for further analysis (Fig. 4C). Six modules were selected from the NLAN, and three modules were from PLAN (Additional file 1: Table S1). Characteristics of each module were visualized with box plots, including the expression of lncRNAs (Additional file 1: Fig. S1A), node degree (Additional file 1: Fig. S1B), clustering coefficient (Additional file 1: Fig. S1C), and betweenness centrality (Additional file 1: Fig. S1D).

### LncRNAs within the modules are highly associated

**Fig. 5:**
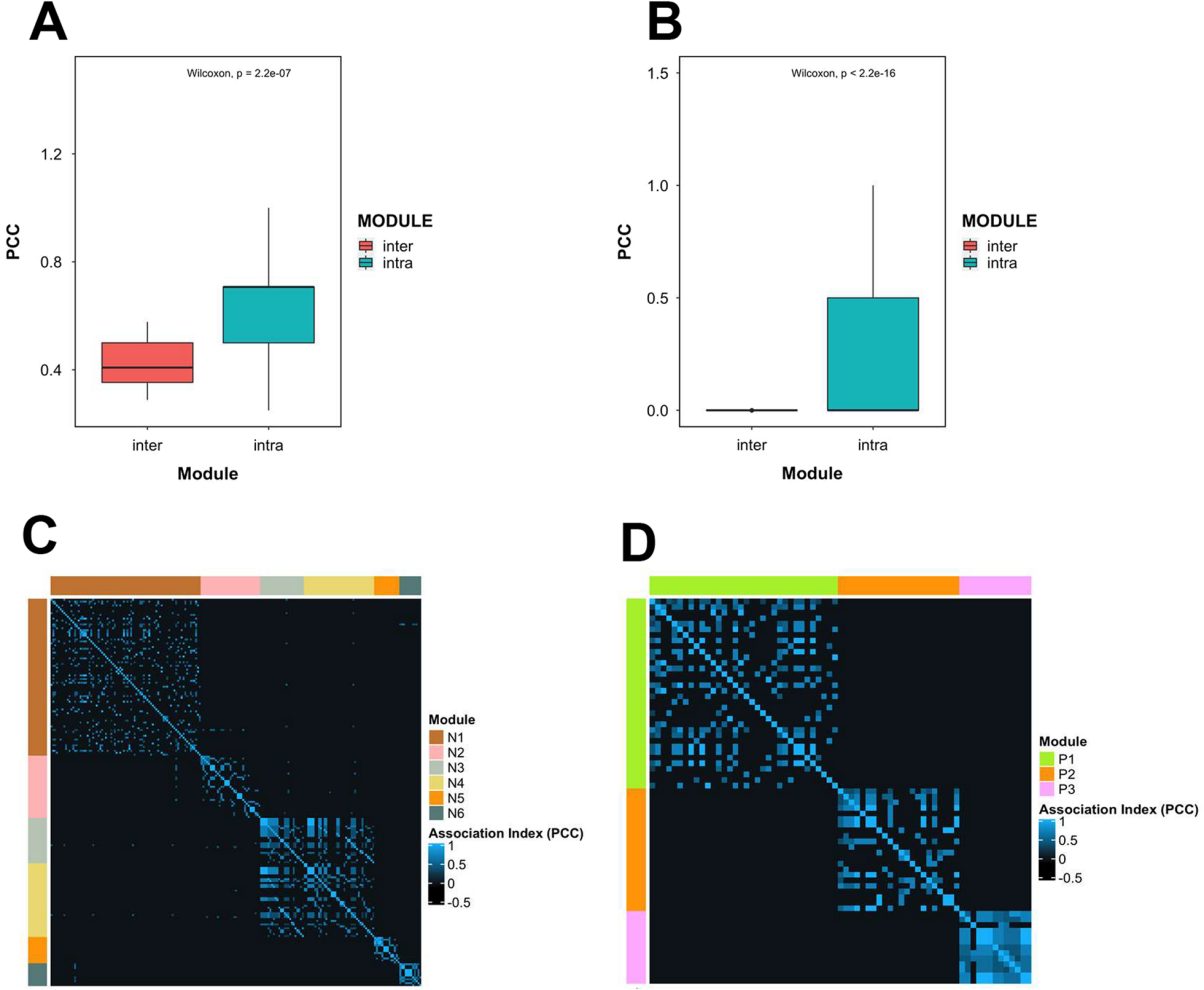
Comparison of intra-module lncRNAs and inter-module lncRNAs. **A**, **B** The PCC between intra-modular lncRNAs and inter-modular lncRNAs were compared in NLAN and PLAN respectively. Wilcoxon test was used to compare two groups. **C**, **D** The PCCs of each lncRNA were visualized in heatmaps. The clusters in heatmpas were highly correspond to lncRNA modules identified in the association networks

Since we postulated that modules are composed of highly associated lncRNAs, we compared the association index, PCC, between intra- and inter-module lncRNAs. It was found that intra-module PCCs are significantly higher than that in inter-module PCCs (Fig. 5A, B), implying lncRNAs within the modules are more alike. The PCC of each lncRNA pair was then visualized with a heat map (Fig. 5C, D). It can be seen that lncRNAs within the same modules are highly associated. The above findings suggested that the association index is can group highly associated lncRNAs together as modules.

### LncRNA modules are associated with the prognosis of LUAD patients

**Fig. 6:**
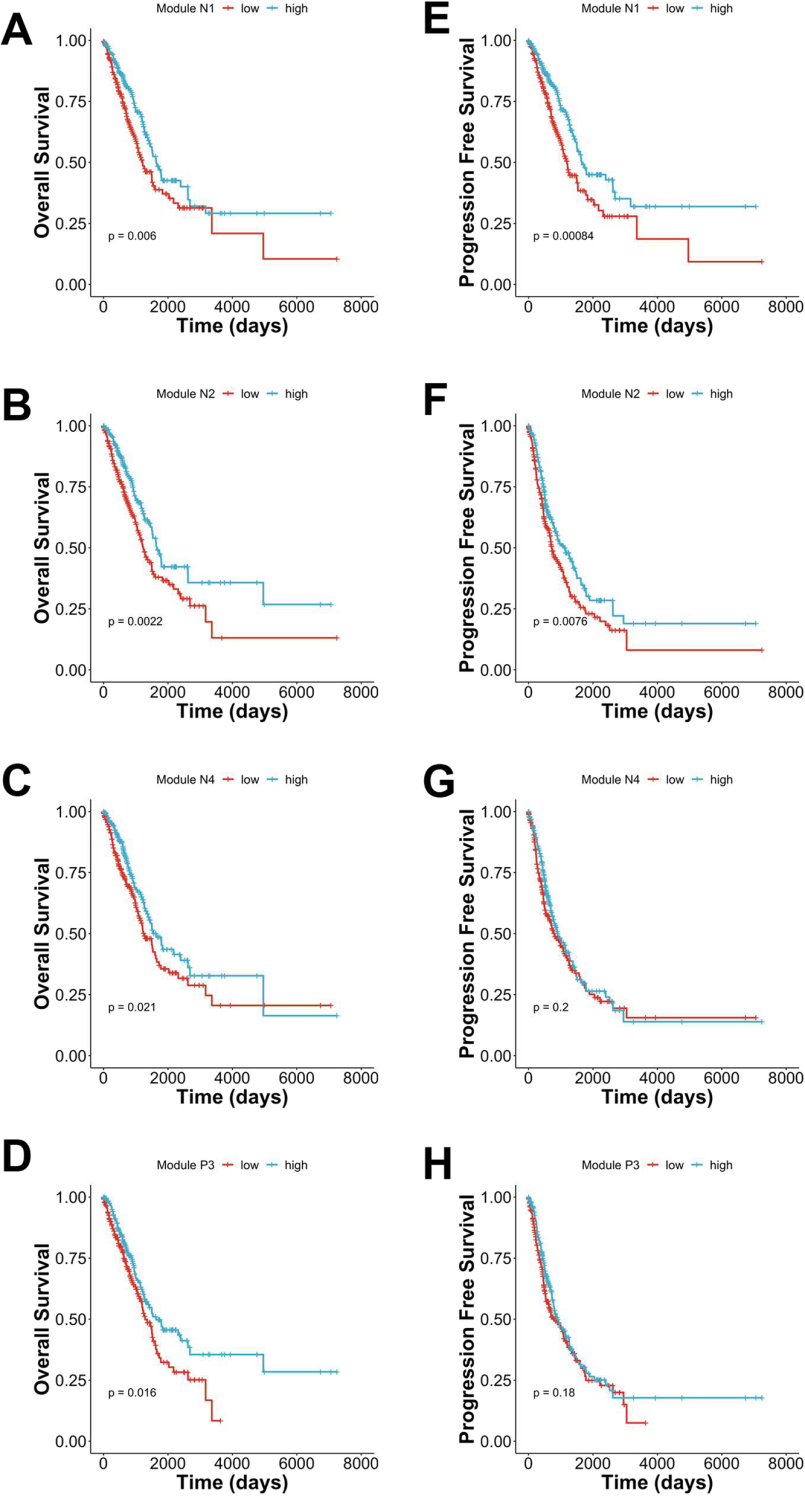
Survival analysis of prognostic lncRNA modules. The median of modular gene expression was used to assign patients into high or low expression groups. Log-rank test was used to test statistical significance. **A**–**D**. Kaplan–Meier curve of the overall survival (OS) in module N1, N2, N4, and P3. **E**–**G**. Kaplan–Meier curve of the event-free survival (EFS) in module N1, N2, N4, and P3

**Fig. 7:**
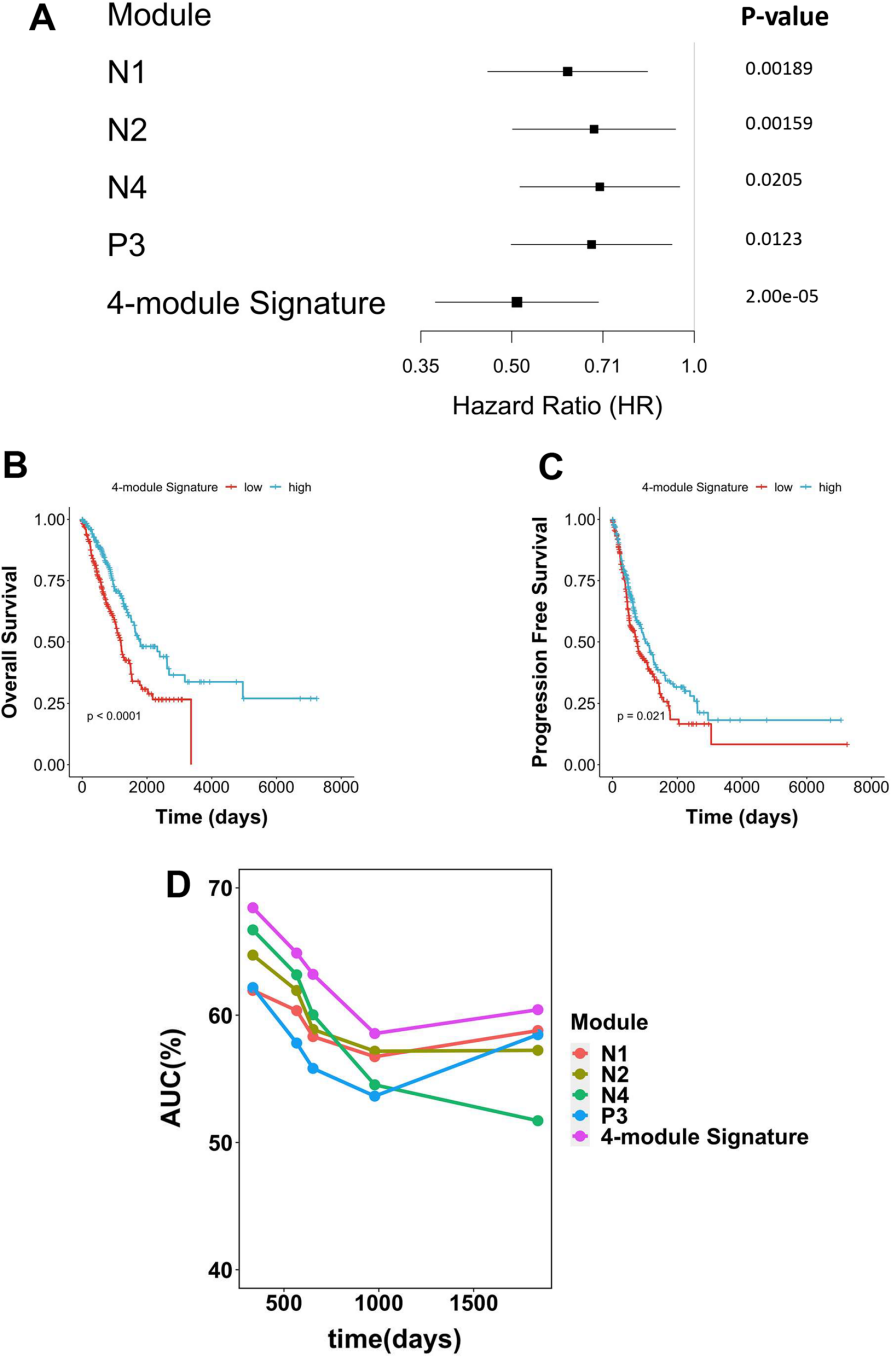
Evaluation of multi-module lncRNA signature. **A** A forest plot showing the HR and p-value under multivariable Cox PH model to assess overall survival of LUAD patients with high *v.s.* low expression of the four lncRNA modules (N1, N2, N4, P3) and the 4-module signature. Age and TNM stage were adjusted. **B** Kaplan–Meier curve of the OS in the 4-module Signature. Log-rank test was used. **C** Kaplan–Meier curve of the EFS in the 4-module Signature. Log-rank test was used. **D** The comparison of AUC of module N1, N2, N4, P3 and the 4-module signature in five different time points

To confirm the effects of the modules on patients’ prognosis, we firstly screened the lncRNAs in the selected nine modules by univariate survival analysis (log-rank test). Because the results from univariate analysis may be confounded by factors that affect the outcome, we next utilised multivariate Cox proportional hazard model to adjust the cancer stage and the age of patients. Afterwards, we excluded the survival-related lncRNAs that were cancer-stage independent or not differentially expressed between cancer and normal tissue (see Additional file 1: Fig. S2). Finally, nine prognosis-related lncRNAs were selected (Table 1). Specifically, there are five prognostic lncRNAs (AC073655.2, AL031775.2, AL122010.1, AC079210.1, and AL391834.2) in module N1, two lncRNAs (AL683813.2 and AC005759.1) in N2, one lncRNA (AC008937.3) in N4, and one lncRNA (AC099343.4) in P3. The modular expression was calculated by summing the coefficient-adjusted expression of the prognostic lncRNA(s). These four lncRNA modules are significantly associated with overall survival (OS) as shown in the Kaplan–Meier plots (Fig. 6A–D). We also inspected the impact of lncRNA modules on patients’ progression free survival (PFS) (Fig. 6E–G). When adjusting the effects of cancer stage and patients’ age on survival, the association between OS and lncRNA module is still significant. (Fig. 7A). Next, since combining multiple lncRNAs into one signature can better predict the outcomes [13, 14, 16], four lncRNA modules (N1, N2, N4, and P3) were pooled into one lncRNA signature. The OS (*p* < 0.0001) and PFS (*p* = 0.021) are significant (Fig. 7B, C). The hazard ratio of the 4-module signature in the multivariable model is 0.51 (95% CI = 0.37, 0.69), and *p* value = 2.00e−05 (Fig. 7A).

**Table 1:** Survival-related lncRNAs in each prognostic lncRNA module and the overall survival

ID	Name	Module	Survival
ENSG00000258365	AC073655.2	N1	Favourable
ENSG00000272402	AL031775.2		Favourable
ENSG00000230163	AL122010.1		Favourable
ENSG00000267461	AC079210.1		Favourable
ENSG00000273226	AL391834.2		Favourable
ENSG00000232611	AL683813.2	N2	Favourable
ENSG00000268650	AC005759.1		Favourable
ENSG00000271828	AC008937.3	N4	Favourable
ENSG00000271646	AC099343.4	P3	Favourable

### Assessment of the lncRNA modular signature as a novel prognostic biomarker in LUAD

To assess the lncRNA modules as a prognostic biomarker in LUAD, we interrogated patients’ OS and compared the AUC at several time intervals (Fig. 7D). The results were consistent with the former survival analyses. The 4-module lncRNA signature is better than individual lncRNA modules at all time points. The highest AUC of the 4-module lncRNA signature appears on the 336th day (AUC = (0.68)).

### The lncRNA modules are related to cancer hallmarks

**Fig. 8:**
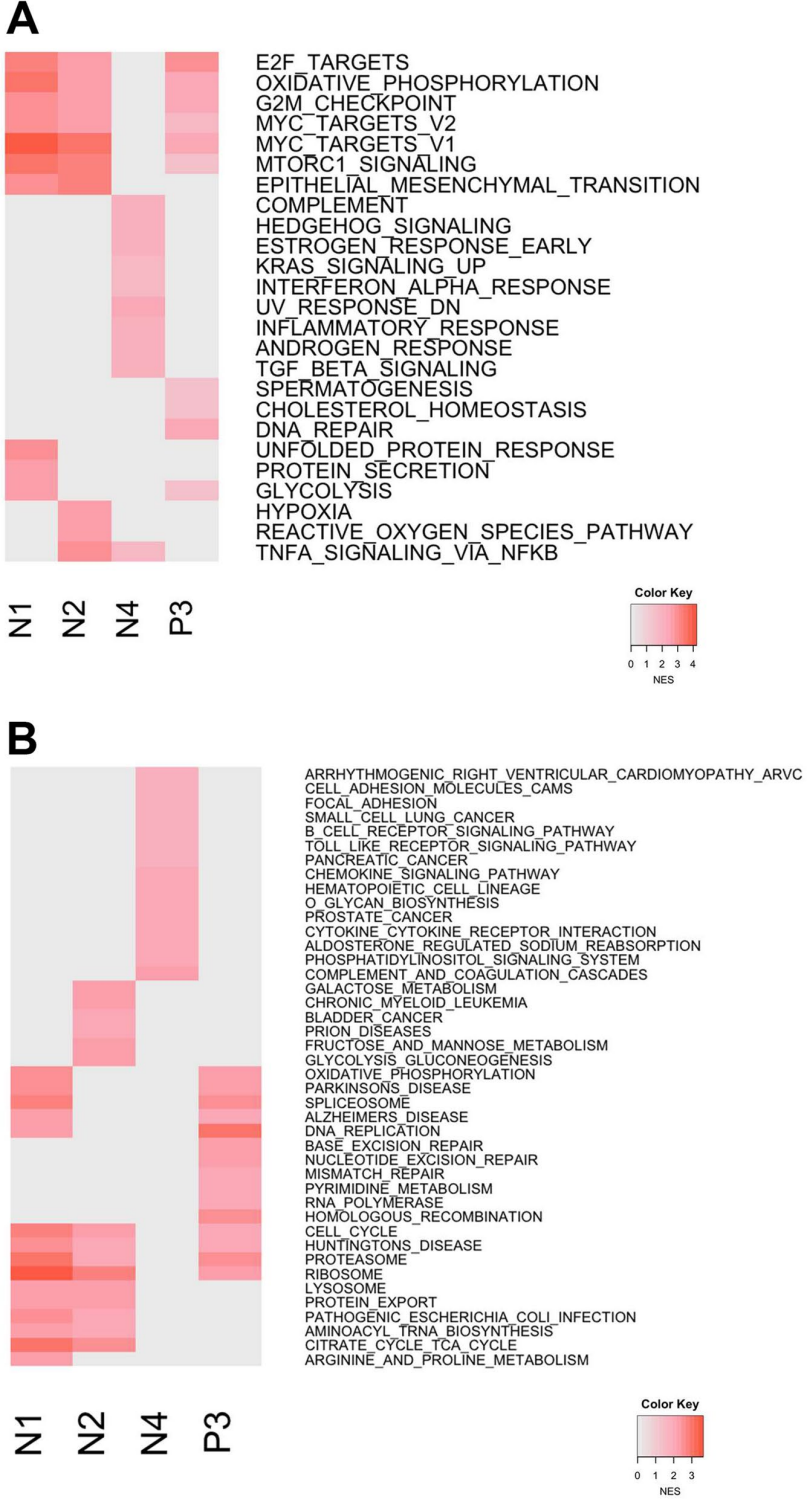
Comparing functions of prognostic lncRNA modules. **A** GSEA was used to conduct functional enrichment analysis where top 10 biological processes, based on normalized enrichment score (NES), in each module were shown (FDR q-value < 0.001). **B** Biological functions regarding KEGG pathways were investigated. Terms with top 15 NES were shown in each module (FDR q-value < 0.001)

Until recently, functions of many lncRNAs in cancer have still not been fully characterized [22]. We used pre-ranked gene set enrichment analysis to deduce the functions of the prognostic lncRNA modules [23]. It was found that Myc targets, E2F targets, and cell cycle were enriched in module N1, N2 and P3. Further, in module N4, inflammation/immunity-related terms, androgen/oestrogen response, and KRAS signalling were revealed. Of note, several pathways of other cancer types were enriched in the module P3, implying the involvement of other cancer types as well (Fig. 8). Some of our enrichment results were consistent with the findings validated in other published literatures, where the expression of the lncRNAs were linked to cancer-related processes such as E2F [24], c-Myc [25, 26], androgen receptor [27, 28] and oestrogen receptor [29]. All in all, the enrichment analysis revealed cancer hallmarks in which lncRNA modules involve. The enriched terms not only reproduce the characters of lncRNAs found previously, but as well reveal several novel mechanisms in which lncRNA modules may participate.

## Discussion

This study utilized an association network approach to group similar lncRNAs, identify four prognosis-related lncRNA modules and assess the performance of the modular signature as a novel biomarker in LUAD. Biologically, these lncRNA modules involve in various oncogenic mechanisms. Altogether, this study not only revealed the roles of lncRNA modules but also came up with a new module-based biomarker in LAUD.

The genetic regulation of lncRNA is complex. Thus far, we merely made a correlation and association analyses to model the relation between lncRNAs and mRNAs. It may not be proper to infer the causal relationship between lncRNAs and mRNAs from this study. In fact, lncRNA can regulate mRNAs through cis- and trans- regulation, while mRNAs can also regulate the expression of lncRNAs through other mechanisms [30, 31]. The direction of regulation between lncRNAs and mRNAs still need further investigation in the future.

Numerous studies also focused on the issue regarding the competing endogenous RNA (ceRNA) networks [32–34]. Via constructing a lncRNA–miRNA–mRNA ceRNA network, the molecular mechanisms of certain diseases can be further clarified. A LUAD-specific ceRNA network revealed that seven lncRNAs were associated with the prognosis through interacting with one miRNA (hsa-mir-31) and 16 mRNAs [35]. *Zhang *et al. identified a LUAD-specific ceRNA network through curating the experiment-supported databases. Based on this ceRNA network, they used random walking and restart algorithm to rank lncRNAs that are associated with LUAD. An in vitro validation was subsequently conducted to prove their prediction that MAPKAPK5-AS1, a novel LUAD-related lncRNA, plays crucial roles in tumour growth [36]. Compared with the construction of ceRNA network in LUAD, our approach mainly used the correlation between lncRNAs and mRNAs to infer the association network of lncRNAs, without considering the roles of miRNAs. However, the advantages of our study include using the real-world data to assess the impact of the lncRNA signature on patients’ survival, and evaluating the lncRNA signature as a biomarker in LUAD.

Network biology has become popular in biomedical researches. Analysing networks can model the complex interactions of the biomolecules, providing more accurate predictions [19, 21, 37–41]. Furthermore, it was revealed that networks can delineate dissimilar molecules and gather similar nodes as modules [42, 43]. Therefore, in this study, we conjectured that similar lncRNAs would co-appear as modules and are highly associated. We further tested the biological functions of these generic modules, and discovered module-based biomarkers in LUAD. It is not denying that surveying the biomarkers within the lncRNA association networks may miss the prognosis-related lncRNAs that are not appear in the networks. However, the goal of this study was to construct a LUAD-specific lncRNA association network and assess whether these modules play important roles in LUAD, instead of revealing all lncRNAs related to the prognosis in LUAD.

One of the future applications of this study is that the distance of modules in the networks can be quantified by the shortest path length (proximity), which was proved to be a successful method to judge the relatedness between clusters of nodes [38]. This fact can be further applied to the cancer drug repurposing. For instance, a recent study found that the proximity between disease-related proteins and drug targets in the protein interactome is an effective measure to predict the response to the drugs [39]. The accuracy of the proximity method even outweigh the traditional expression-based perturbation methods, such as The Library of Integrated Network-Based Cellular Signatures (LINCS) [39]. In reality, according to the latest LUAD guideline [3], targeting lncRNA modules is not feasible. Therefore, the drug repurposing method that interrogating the proximity between lncRNA module-related proteins and drug targets may be one of the solutions.

We referred our findings to The Cancer LncRNome Atlas [44], a database collecting lncRNA alterations across multiple human cancer types (http://fcgportal.org/TCLA/search.php). It can be seen that the lncRNAs revealed in this study were novel. Copy number variation (CNV) deletion were found in AC073655.2 and AC099343.4, but the alterations in expression level have been unclear. Four lncRNAs (AC008937.3, AL683813.2, AL031775.2, AL122010.1, and AC005759.1) were detectable in LUAD. The other two lncRNAs, AL391834.2, and AC079210.1, were not detectable in LUAD (Additional file 1: Table S2). It is believed that gene expression can be affected by CNV, where decreased gene expression may be attributed to deletions [45]. In our study, we found that decreased expression of AC073655.2 and AC099343.4 are associated with poorer prognosis in LUAD patients. Therefore, it may not be inappropriate to postulate that the underlying mechanisms might be associated with the deletions. However, further investigations will be needed to validate this hypothesis.

## Conclusions

In conclusion, by mining the expression profile from TCGA, we constructed lncRNA-association networks and revealed four crucial LUAD-specific lncRNA modules. These modules involve in several cancer-related biological mechanisms and affect patients’ survival. The 4-module lncRNA signature can be used as a prognostic biomarker for LUAD. As far as we know, this is the first study using lncRNA association network to discover promising lncRNA modular biomarkers in LUAD. This method offered a novel insight into the co-regulated lncRNAs in lung cancer and may become a framework for future studies, such as the lncRNA module-based target therapy.

## Methods

### Pre-processing of lncRNA and mRNA expression profiles

We retrieved the transcriptome data of lung adenocarcinoma (LUAD) from Genomic data commons (GDC) data portal. The lncRNA and mRNA expression profile were collected from the HTSeq-FPKM-UQ workflow of TCGA-LUAD project (n = 553) in February 2018. The pre-processing procedure of the gene expression profile was adjusted from our previous work [21]. In brief, genes with more than 20% of values to be NAs were removed. Next, the remaining NAs were filled with 0. We then adjusted the expression of genes with log2 transformation. To link the Ensembl gene ID with gene type, we used Ensembl 92 (GRCh38.p12) as the reference genome. Finally, The Ensembl gene ID was converted to gene symbol by referring to BioMart database [46]. The pre-processed gene expression file contained 4342 lncRNAs and 16618 mRNAs.

We also used identical pre-processing steps to gather the expression profile of adjacent normal tissue for subsequent analyses. These samples were used as control group and pooled from the adjacent normal tissue in both LUAD and lung squamous cell carcinoma (LUSC) project from TCGA, consisting of 108 samples in total.

### Identification of LncRNA-correlated mRNAs

To model the relation between lncRNAs and mRNAs, we used Spearman’s correlation coefficient (SCC) (1) to quantify the correlation of lncRNA and mRNA.

1 $$SCC = 1 - \frac{{6\sum d_{i}^{2} }}{{n\left( {n^{2} - 1} \right)}}$$

where d_i_ = the difference between the two ranks of each investigated gene, and n = sample size.

The SCCs were then transformed to mutual rank (MR) index. The MR is the geometric average of the SCC rank [21]. That is, for the gene pair lncRNA *X* and mRNA *Y*, the mutual rank is defined as follow:

2 $$MR_{XY} = \sqrt {Rank_{X \to Y} \times Rank_{Y \to X} }$$

In order to select negatively and positively lncRNA-correlated mRNAs, we ranked the SCCs in ascending and descending order to represent negatively and positively correlated mRNAs respectively. We then defined lncRNA-mRNA pairs with top one percent of MR index as highly correlated lncRNA-mRNA pairs.

### Construction of lncRNA association networks

We used the lncRNA mRNA correlation bi-partite networks to deduce the association between lncRNAs. Pearson correlation coefficient (PCC) (3), an association index, was computed to define the association between each lncRNA.

3 $$PCC_{XY} = \frac{{\left| {{\text{N}}\left( {\text{X}} \right) \cap {\text{n}}\left( {\text{Y}} \right)} \right|n_{y} - \left| {{\text{N}}\left( {\text{X}} \right)} \right|\left| {{\text{N}}\left( {\text{Y}} \right)} \right|{ }}}{{\sqrt {\left| {{\text{N}}\left( {\text{X}} \right)} \right|\left| {{\text{N}}\left( {\text{Y}} \right)} \right|\left( {n_{y} - \left| {{\text{N}}\left( {\text{X}} \right)} \right|} \right)\left( {n_{y} - \left| {{\text{N}}\left( {\text{Y}} \right)} \right|} \right)} }}$$

where N(X) is the number of mRNAs correlated with lncRNA X, N(Y) is the number of genes correlated with lncRNA Y, n_y_ is the number of mRNAs correlated with both lncRNA X and lncRNA Y. We defined two lncRNAs being associated if and only if they shared PCC > 0.7. We then collected lncRNA pairs with this criterion into a lncRNA-lncRNA edge list and visualized the lncRNA association network with Cytoscape [47]. The lncRNA association network constructed from negatively correlated mRNAs were coined as negative lncRNA association network (NALN). On the other hand, those considered positively correlated mRNAs were termed positive lncRNA association network (PLAN).

### Survival analysis and biomarker assessment

To select lncRNA modules that determine prognosis of LUAD patients, we designed a three-step selection procedure, implemented with the R package: *survival* (https://CRAN.R-project.org/package=survival) and *survminer* (http://www.sthda.com/english/rpkgs/survminer/). Firstly, the survival time, age at diagnosis, TNM stage, event type (cancer progression or death), and censoring information of TCGA LUAD were collected from clinical data in GDC data portal. We divided patients into two subgroups based on the median of lncRNA expression. The univariate survival analysis was conducted by log-rank test. Secondly, to adjust known confounding factors, including patients’ age and cancer stage, the genes that passed log-rank test were subsequently examined by multivariable Cox-proportional hazard model. Finally, the remaining lncRNAs without stage-dependency or differential expression were discarded. The stage-dependency analysis was conducted by comparing lncRNA expression across different TNM stage (from stage I to stage IV). Kruskal–Wallis test was used for hypotheses testing. For the differential expression analysis, we compared the gene expression between cancer and adjacent normal tissue with Wilcoxon test.

For the biomarker assessment, time-dependent area under the receiver operating characteristic (time-AUC) was calculated by using the R package ‘time ROC’ [48].

### Functional gene set enrichment analysis

To investigate functions of prognostic lncRNA modules, we interrogated lncRNA-correlated mRNAs to conduct pre-ranked GSEA [23]. The mRNAs were ranked based on the correlation (MR index) with the lncRNAs within each module. In PLAN, since the positively correlated mRNAs were more important, the SCCs were arranged in the ascending order, from small to large, which led to higher MR index in the positively correlated mRNAs. By contrast, SCCs were arranged in descending order, which resulted in higher MR index in negatively correlated mRNAs. Gene sets database, Hallmark and KEGG pathway, from MSigDB v7.1 [49, 50] were selected. The chip platform was from Human_ENSEMBL_Gene_MSigDB.v7.1.chip. The top 10 and 15 were selected from Hallmark and KEGG pathway respectively. All selected terms were significant, with FDR-q value < 0.001.

## Supplementary Information

**Additional file 1.** Detailed components in each lncRNA module.

## Data Availability

The RNA-seq transcriptome profiling of lung adenocarcinoma from TCGA is freely available via GDC data portal (https://portal.gdc.cancer.gov/).

## Abbreviations

LncRNA: Long non-coding RNA
LUAD: Lung adenocarcinoma
TCGA: The Cancer Genome Atlas
HR: Hazard ratio
CI: Confidence interval
AUC: Area under the receiver operating characteristic curve
EMT: Epithelial-mesenchymal transition
SCC: Spearman’s correlation coefficient
MR: Mutual rank
PCC: Pearson correlation coefficient
LAN: LncRNA association network
PLAN: Positive lncRNA association network
NALN: Negative lncRNA association network
OS: Overall survival
PFS: Progression free survival
ceRNA: Competing endogenous RNA
LINCS: The Library of Integrated Network-Based Cellular Signatures
CNV: Copy number variation
