# Supplementary material for: Modular signature of long non-coding RNA association networks as a prognostic biomarker in lung cancer
Source: BMC Med Genomics. 2021 Dec 6;14(Suppl 3):290. doi: 10.1186/s12920-021-01137-0 (PMC8650235; doi:10.1186/s12920-021-01137-0)
Supplement: Supplementary file 1 — Additional file 1. Detailed components in each lncRNA module. [file 12920_2021_1137_MOESM1_ESM.docx]

**Supplementary information**

**Modular signature of long non-coding RNA association networks as a prognostic biomarker in lung cancer**

Albert Li, Wen-Hsuan Yu, Chia-Lang Hsu, Hsuan-Cheng Huang, Hsueh-Fen Juan

**Supplementary information**

**Additional file 1: Table S1**. Detailed components in each lncRNA module.

**Additional file 2: Figure S1**. Characteristic of generic lncRNA modules.

**Additional file 3: Figure S2**. Identification of prognostic lncRNAs from lncRNA association network.

**Additional file 4: Table S2**. The expression and CNV alteration of the lncRNAs collected in The Cancer LncRNome Atlas.

**Additional file 1: Table S1**. Detailed components in each lncRNA module.

| Module | lncRNAs |
| --- | --- |
| N1 | AP001157.1, AC073655.2, AC010300.1, AC010883.1, AC006480.2, AC135178.5, AL159169.2,AC073592.1, AC009318.1, GABPB1-AS1, AC007038.2, AC015727.1, AC021739.2, AC010538.1, AC009107.2, AL021707.1, AL031775.2, AC073389.3, AC004594.1, AL031716.1, AL359921.1, AC004076.2, PSPC1-AS2, AL669831.5, AL450384.2, AL117379.1, SRRM2-AS1,U73166.1, AC106820.3, AL031709.1, AC004466.1, AL513218.1, AL122010.1, AL162274.2, AL096701.3, AC232271.1, AC087392.3, AC124319.3, AL590822.2, AC003102.1, AC126118.1, AC010491.1, AL121895.1, AC013468.1, AP001267.3, AC092809.4, CAMTA1-DT, AC063948.1, AL137802.2, AL117336.3, AC026191.1, U91328.3, AL589765.6, AC092757.3, AC114939.1, AC092794.1, AP003717.1, AL391834.2, AC079210.1, AC002550.2, AL731563.3, AC002553.2, AC068620.2, AL354760.1, AC087500.2, AC006435.2, AC027601.3, AC022007.1, AC116914.2, AP001160.1, AL136295.6, AL021392.1, AC079174.2, AC026471.4, AL359265.3, AC009120.5, HCG27, AC087752.4, AC093726.2, AC073575.2, AC087742.1, AL031705.1, AC096642.1 |
| N2 | AC004039.1,AP002907.1,AC106820.5,AP001893.1,DCUN1D2-AS,AC004877.1,AC245052.4,AC087222.1,AL139241.1,AL121987.2,AC138932.5,SSBP3-AS1,EDRF1-AS1,AC104785.1,ZNF32-AS2,AL021707.7,AC245884.8,AC245060.5,AC004466.3,AC008764.8,AL683813.1,LINC01534,AC005759.1,AC018638.7,AL513327.3,AC018766.1,AC138956.2,AC109460.1,AL928654.2,AC027682.4,AL157392.4,AC092301.1 |
| N3 | AL035448.1,EFCAB14-AS1,AC010761.3,AC002128.2,AC074138.1,AL590729.1,LINC01355,AP003096.1,AC009095.1,AC008731.1,AL450306.1,AP001020.3,AP001001.1,AC080162.1,AL365277.1,AC097634.1,AC104534.1,CR936218.1,MALAT1,AC090337.1,AC069549.1,AL513365.2,AC006504.1,Z98885.3 |
| N4 | AC007336.1,AC110792.3,AC008937.3,AC009163.7,MCCC1-AS1,SMC2-AS1,AC007566.1,SEMA6A-AS1, AC136604.2,LINC02062,AC119403.1,AC034236.2,AL035563.1,AC012313.6,AC007686.3 |
| N5 | AC010719.1,AL158071.4,ZNF252P-AS1,AC092123.1,AC084824.5,AC048341.2,AC007879.3,AC074044.1,AC211476.2,AD000671.3,AC011444.1,AC004253.1,AP001330.5,AC005899.6 |
| N6 | AC009283.1,AC133919.2,AC008764.6,AC087239.1,AC093151.2,AC104463.2,AL008582.1,VASH1-AS1,AL136304.1,LINC00685,AC092118.2,AC005332.1 |
| P1 | AL669831.5,AL365330.1,CAPN10-AS1,AC073389.3,AP001157.1,AL731569.1,MIR4453HG,AC073195.1,LINC00115,CAHM,AC023355.1,AC025178.1,LINC01004,STAM-AS1,AL162741.1,AC211476.2,AC006252.1,AC017083.1,AL513218.1,ZNF252P-AS1,AP002907.1,AC021321.1,NFYC-AS1,AL008582.1,AC008764.6,AL121658.1,AC013731.1,AL139289.1,AP001107.1,KDM4A-AS1,AC107375.1,AC009148.1,AC132192.2,AC079322.1 |
| P2 | AC008731.1,AC011825.2,AP001793.1,AL133406.2,AC027514.2,AC080162.1,AL133230.1,PITRM1-AS1,AC018766.1,Z98885.3,FLNB-AS1, AC011466.3,AC064801.1,AC092301.1,AC131971.1,AC245060.6,AC053513.1,AC138932.5,AC007216.4,AC009095.1,AC024267.4,AL035448.1 |
| P3 | AC073389.1,AC010359.1,AC099343.3,AC026304.1,AL135925.1,AL132639.2,LINC01144,ENO1-AS1,AC127070.1,AP004609.3,AC012313.9,CAMTA1-DT,AC004812.2 |

**Additional file 2: Figure S1**. Characteristic of generic lncRNA modules.

Figure S1. Characteristic of generic lncRNA modules. (A) The median lncRNA expression in each module across samples. (B) The degree distribution in each module across samples. (C) The betweenness centrality in each module across. (D) The clustering coefficient in each module across samples.

**Additional file 3: Figure S2**. Identification of prognostic lncRNAs from lncRNA association network.

Figure S2. Identification of prognostic lncRNAs from lncRNA association network. The trend of gene differential expression, cancer stage dependency and KM curve were compared to select prognostic lncRNAs. Five prognostic lncRNAs were found in (A), two prognostic lncRNAs were found in (B), one prognostic lncRNA was found in (C), and one prognostic lncRNA was found in (D).

| **Name** | **Detectable (expression)** | **CNV alteration** |
| --- | --- | --- |
| AC073655.2 | NA | 12q24.33: deletion |
| AL031775.2 | Yes | NA |
| AL122010.1 | Yes | NA |
| AC079210.1 | NA | NA |
| AL391834.2 | NA | NA |
| AL683813.2 | Yes | NA |
| AC005759.1 | Yes | NA |
| AC008937.3 | Yes | NA |
| AC099343.4 | Yes | 4q35.1: deletion |

**Additional file 4: Table S2**. The expression and CNV alteration of the lncRNAs collected in The Cancer LncRNome Atlas.
